# Long-lasting insecticidal net use and asymptomatic malaria parasitaemia among household members of laboratory-confirmed malaria patients attending selected health facilities in Abuja, Nigeria, 2016: A cross-sectional survey

**DOI:** 10.1371/journal.pone.0203686

**Published:** 2018-09-13

**Authors:** Amaka Pamela Onyiah, IkeOluwapo O. Ajayi, Hannah O. Dada-Adegbola, Babatunde O. Adedokun, Muhammad S. Balogun, Patrick M. Nguku, Olufemi O. Ajumobi

**Affiliations:** 1 Nigeria Field Epidemiology and Laboratory Training Programme, Abuja, FCT, Nigeria; 2 Department of Epidemiology and Medical Statistics, University of Ibadan, Ibadan, Oyo State, Nigeria; 3 Department of Medical Microbiology and Parasitology, University of Ibadan, Ibadan, Oyo State, Nigeria; 4 National Malaria Elimination Programme, Abuja, FCT, Nigeria; George Washington University School of Medicine and Health Sciences, UNITED STATES

## Abstract

**Introduction:**

In Nigeria, malaria remains a major burden. There is the presupposition that household members could have common exposure to malaria parasite and use of long-lasting insecticidal net (LLIN) could reduce transmission. This study was conducted to identify factors associated with asymptomatic malaria parasitaemia and LLIN use among households of confirmed malaria patients in Abuja, Nigeria.

**Methods:**

A cross-sectional survey was conducted from March to August 2016 in twelve health facilities selected from three area councils in Abuja, Nigeria. Participants were selected using multi-stage sampling technique. Overall, we recruited 602 participants from 107 households linked to 107 malaria patients attending the health facilities. Data on LLIN ownership, utilization, and house characteristics were collected using a semi-structured questionnaire. Blood samples of household members were examined for malaria parasitaemia using microscopy. Data were analyzed using descriptive statistics, Chi-square, and logistic regression (α = 0.05).

**Results:**

Median age of respondents was 16.5 years (Interquartile range: 23 years); 55.0% were females. Proportions of households that owned and used at least one LLIN were 44.8% and 33.6%, respectively. Parasitaemia was detected in at least one family member of 102 (95.3%) index malaria patients. Prevalence of asymptomatic malaria parasitaemia among study participants was 421/602 (69.9%). No association was found between individual LLIN use and malaria parasitaemia (odds ratio: 0.9, 95% confidence interval (95%CI): 0.6–1.3) among study participants. Having bushes around the homes was associated with having malaria parasitaemia (adjusted OR (aOR): 2.7, 95%CI: 1.7–4.2) and less use of LLIN (aOR: 0.4, 95%CI: 0.2–0.9). Living in Kwali (aOR: 0.1, 95% CI: 0.0–0.2) was associated with less use of LLIN.

**Conclusion:**

High prevalence of asymptomatic malaria and low use of LLIN among household members of malaria patients portend the risk of intra-household common source of malaria transmission. We recommend household health education on LLIN use and environmental management. Study to explore the role of preventive treatment of household members of confirmed malaria patient in curbing transmission is suggested. Strategies promoting LLIN use need to be intensified in Kwali.

## Introduction

Malaria is transmitted by the bite of infected female anopheles mosquitoes and most of the bites occur indoors [1]. This constitutes a risk of trans-infection among household members as they are exposed to similar environmental conditions. A household where one individual has been diagnosed to have malaria is therefore more likely to have a cluster of infected individuals [2,3]. Malaria infection outcomes can be asymptomatic, uncomplicated or severe. Asymptomatic malaria remains a challenge to malaria elimination as asymptomatic gametocyte carriers contribute to persistence of malaria transmission [4]. The World Health Organisation (WHO) has recommended that people of all ages sleep under an insecticide-treated net (ITN) to prevent continued transmission of malaria by mosquitoes [5].

Approximately 3.2 billion people are at risk of malaria infection globally [6]. The burden of malaria is greatest in fifteen countries in sub-Saharan Africa which account for an estimated 80% of all malaria deaths [7]. In 2016, Nigeria accounted for about 27% of the global burden of malaria infections and 30% of mortality [7]. Strategic interventions for malaria control in the country include case management of malaria using artemisinin-based combination therapies (ACTs), integrated vector management (IVM) including use of ITNs, prevention and treatment of malaria in pregnancy (MIP) and other health system strengthening intervention strategies. To achieve universal LLIN coverage and effective malaria control, a strategy of scaled-up mass distribution of free long-lasting insecticidal nets (LLIN) was embarked upon in 2009 across the 36 states and Federal Capital Territory of the country [8]. However, the 2015 National Malaria Indicator Survey found LLIN utilization to be sub-optimal at 37.2%.

In Nigeria, there is paucity of information on factors associated with LLIN use and asymptomatic malaria parasitaemia among all age groups. Currently, surveillance for asymptomatic malaria parasitaemia which remains the reservoir for persistent transmission, is not yet a priority in Nigeria despite the envisioned goal of malaria elimination. Therefore, we conducted a study to determine prevalence of asymptomatic malaria parasitaemia, determine the relationship between LLIN use and asymptomatic malaria parasitaemia, assess contributing factors to malaria infections, and identify factors associated with LLIN use among household members of confirmed malaria patients in FCT, Abuja, Nigeria. The rationale for selecting households where one was already diagnosed for malaria was to establish possible common exposure.

## Methods

### Study area

The study was conducted from March to August 2016 in Federal Capital Territory (FCT), Abuja, Nigeria, a city with a total projected population of 3,564,100 in 2016 [9] distributed across six area councils. Abuja Municipal area council, one of the six area councils, is the metropolis with a projected population of 1,967,500 in 2016. FCT is located in the north-central geo-political zone. There is a humid rainy season from April to September with temperature ranging from 22°C to 30°C, a hot dry season from October to March with a temperature of 12°C to 40°C and brief period of harmattan in December. The annual total rainfall ranges from 1100mm to 1600mm and altitude is 476m. Malaria is endemic in the area. As a rapidly growing capital city, almost all tribes in Nigeria are represented in Abuja.

There are 3 tertiary, 14 secondary, and 179 primary public health facilities distributed across the six area councils in the FCT. There are also 673 registered private health facilities and 81 laboratories. The National Malaria Indicator Survey (MIS) conducted in 2015 in all 36 states of Nigeria and FCT showed that north-central zone had the second highest malaria prevalence of 32.0% by microscopy among children aged 6 to 59 months.

### Study design

A cross-sectional household survey was conducted in Abuja Municipal Area, Kuje and Kwali area councils.

### Sample size

Using 36.6% prevalence of malaria parasitaemia from a previous community-based study in Plateau State, north-central Nigeria [10], 5% level of precision, design effect of 1.5 to take care of clustering effect in households and enable us detect any variability, and 10% non-response rate, the calculated minimum sample size (n) was 593 study participants.

### Study population

The subjects were all household members of malaria patients attending selected health facilities and residing in Abuja. Individuals who had not lived in the house for more than 7 days (minimum incubation period of malaria) [6], those who took anti-malarial medicines two weeks prior to the study, those with blood dyscrasias (such as haemophilia and sickle cell disease), and critically ill individuals were excluded from the study.

### Sampling technique

**Fig 1:**
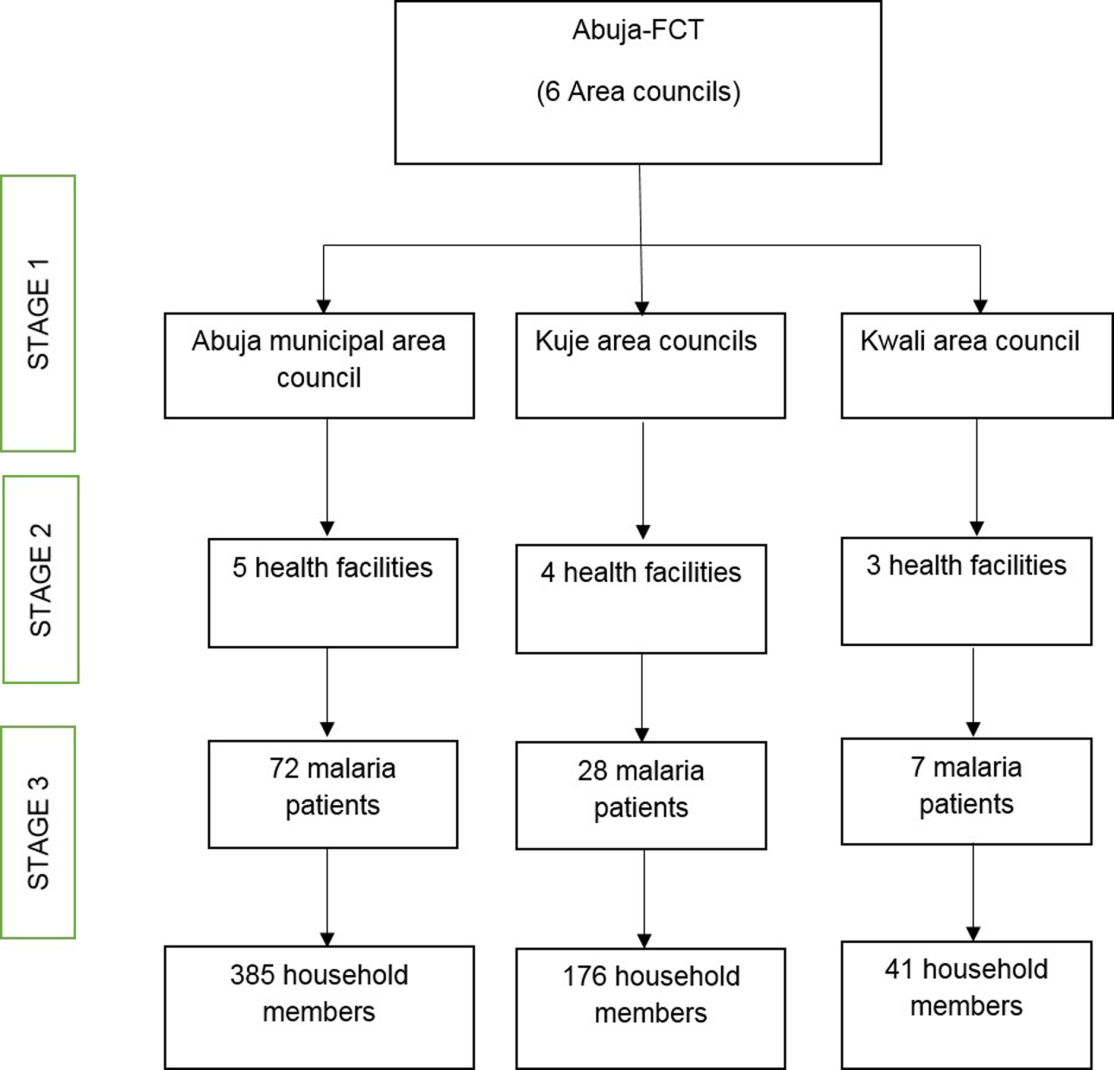
Flow chart of sampling technique.

Fig 1 shows the sampling flowchart. Multi-stage sampling technique was used.

1. Three of the six area councils in the FCT namely Abuja Municipal, Kwali, and Kuje were randomly selected.
2. The sampling frame of all public and private facilities offering malaria microscopy services (which are usually only available at secondary health facilities) in the three area councils was developed. The intention was to select five health facilities per area council but only four and three health facilities met this criterion in Kuje and Kwali area councils respectively. In all, 12 health facilities were selected. Laboratory-confirmed malaria patients were recruited consecutively from each of the selected health facilities until the minimum sample size was reached within the allotted study period.
3. Trained research assistants visited households of the identified malaria patients within 7 days and enrolled all household members who consented to participate in the study.

### Data collection

Pretested questionnaires were interviewer-administered in English to household members of malaria patients. The questionnaire was adapted from that used in 2015 National MIS and it sought information on participants’ socio-demographic and economic characteristics, house characteristics, LLIN ownership and utilization, use of other malaria preventive measures and knowledge of LLIN and other prevention measures. Blood samples of household members were collected by finger/heel prick and tested using malaria rapid diagnostic test (SD BIOLINE Malaria Ag P.f RDT; manufactured in Germany on 2014/09/03; expiry date—2014/09/02) for the qualitative detection of Histidine-rich protein II antigen of malaria *Plasmodium falciparum* in human whole blood. Thick blood films were prepared by trained medical laboratory scientists on a single slide, air dried and stained with Giemsa on the day of collection for microscopy. Blood films were read independently by trained microscopists (with over 10 years of experience in malaria microscopy) using x100 oil immersion objective to determine malaria parasitaemia status and density. The number of asexual parasites per microlitre of blood in a thick film was counted against 200–500 white blood cells (WBCs) and reported in μL using standard number of 8000 leukocytes). If, after counting 200 WBC, 100 or more parasites were found, the results were recorded against number of parasites/200 WBC. If fewer than 100 parasites were found after counting 200 WBCs, parasite quantification continued until 500 WBCs were counted. All parasites in the final field were counted even if the count exceeded 500 WBCs. [11]. Ten percent of the household sample slides and index case slides were rechecked by a second reader, who is also a trained medical laboratory scientist with over 10 years of experience in malaria microscopy, for quality assurance. There was no discordance in ascertaining presence of malaria parasites when slides were read by second reader. Thus, the need for third reader did not arise.

### Study variable definitions

A household was defined as a person or group of persons, related or unrelated, who usually live together in the same dwelling unit, have common cooking and eating arrangements, and acknowledge one adult member as the head of the household. A member of the household is any person who usually lives in the household [12]. Ownership of Long-lasting insecticidal net was defined as having possession of an LLIN, whether or not it is used [13]. Use of LLIN was defined as sleeping under an LLIN the night prior to the survey/interview/visit and asymptomatic malaria parasitaemia was defined as the presence of asexual parasites in the blood without symptoms of illness [14]. Independent variables were age, sex, educational level of respondents, and housing characteristics.

### Data analysis

Data collected were checked for completeness, cleaned for inaccuracies on the field. Data were entered and analyzed using Epi Info 7 software and SPSS. The prevalence of malaria parasitaemia and proportion of those who own and use LLINs were calculated. Factors associated with LLIN use, and factors that may be responsible for malaria transmission in a household were determined using bivariate analysis.Two logistic regression models were constructed using SPSS Generalized Estimating Equations to determine independent predictors of malaria transmission and LLIN use. Codes for area council, health facility, and household were used as within-subject variables to account for the intra-cluster correlation in the data. The level of significance of results was set at 5%.

### Management of tested household members

Individuals with positive results were offered treatment according to national guidelines: artemether-lumefantrine for non-pregnant individuals older than twelve months of age. Individuals younger than twelve months and pregnant women with a positive test were referred to the nearest health facility for evaluation and care.

### Ethics approval and consent to participate

Ethical approval was obtained from the Federal Capital Territory Health Research Ethics Committee (Protocol approval number: FHREC/2015/01/65/09-11-15). Written informed consent was obtained from each participant ≥18 years or from parents of minors (0–17 years), as well as additional verbal assent from minors over the age of 6 years prior to the interview. Confidentiality of the information provided by the participants was ensured. All the interviews were conducted with codes recorded on the questionnaire to represent the names.

## Results

Overall, 602 study participants were recruited from households of 107 parasitologically-confirmed malaria patients (median: 4.5 household members/household, range: 1 to 20 household members/household).

Of the 602 household members recruited, 331 (55.0%) were females and their median age was 16.5 years, with an interquartile range (IQR) of 23 years. Of the 331 females, 5 (1.5%) were pregnant. Most of the participants (78.2%) were Christians and of Igbo ethnicity (30.2%). One hundred and twenty-seven (21.1%) had no formal education. (Table 1).

**Table 1:** Socio-demographic characteristics of household members of malaria patients attending health facilities in Abuja, Nigeria 2016 [N = 602].

Characteristics	Frequency n (%)
**Age-group (years)**	
<5	91 (15.1)
5–9	112 (18.6)
10–19	136 (22.6)
20–24	50 (8.3)
25–34	111 (18.4)
≥ 35	102 (16.9)
**Sex**	
Female	331 (55.0)
**Religion**	
Christian	471 (78.2)
Muslim	131 (21.8)
**Ethnicity**	
Igbo	182 (30.2)
Hausa	118 (19.6)
Gbagyi	77 (12.8)
Yoruba	73 (12.1)
Idoma	30 (5.0)
Igala	25 (4.2)
Others^a^	97 (16.1)
**Educational level**	
None	127 (21.1)
Primary	164 (27.2)
Secondary	182 (30.2)
Tertiary	129 (21.4)

^a^ represents participants from 29 Nigerian tribes not listed above

Seventy-eight percent of the study participants lived in flats and 79.1% of them lived in houses with cement-plastered walls. Thirty-five percent of participants had bushes around their homes while 12.6% had uncovered water receptacles such as gutters and open water containers around their homes (Table 2).

**Table 2:** Characteristics of residences of household members of malaria patients attending health facilities in Abuja, Nigeria 2016 [N = 602].

Characteristics	Frequency n (%)
**House types**	
Block of flats	470 (78.0)
Hut	77 (12.9)
Duplex	30 (5.0)
Bungalow	25 (4.1)
**Wall types**	
Cement-plastered	476 (79.1)
Mud	126 (20.9)
**Bushes around the house**	
Yes	210 (34.9)
No	392 (65.1)
**Uncovered water receptacles around house**	
Yes	76 (12.6)
No	526 (87.4)

Of the 107 households, 47 (43.9%) owned at least one LLIN while 35 (32.7%) had at least one individual who slept under the net the night before the survey. Of the 602 study participants, 148 (24.6%) owned LLINs, of which 81 (54.7%) purchased the LLINs. Of the 148 who owned LLINs, 107 (72.3%) slept under the net the night before the survey.

Table 3 looks at factors associated with LLIN use among those who owned LLINs. Females who used LLINs (75.0%) were slightly more than males who used LLINs (69.4%). There was no significant difference in the use of LLIN among the different age groups. In the bivariate analysis, the odds of using LLIN was lower among those who lived in Kwali area council (OR: 0.1, 95% CI: 0.0–0.2) than those who lived in AMAC (Table 3). After controlling for other variables, having bushes around the house (adjusted odds ratio (aOR): 0.4, 95% CI: 0.2–0.9) and living in Kwali (aOR: 0.1, 95% CI: 0.0–0.2) were found to be predictors of LLIN use (Table 3).

**Table 3:** Predictors of LLIN use among the household members of malaria patients that owned at least one LLIN, Abuja, Nigeria, 2016 [N = 148].

Characteristics	Used LLIN N = 107	Did not use LLIN N = 41	Crude OR (95% CI)	Adjusted OR (95% CI)
	n (%)	n (%)		
**Sex**				
Female	57 (75.0)	19 (25.0)	ref	ref
Male	50 (69.4)	22 (30.6)	0.8 (0.4–1.6)	0.7 (0.3–1.6)
**Age group (years)**				
<5	20 (74.1)	7 (25.9)	ref	ref
5–9	22 (75.9)	7 (24.1)	1.1 (0.3–3.7)	1.7 (0.2–15.1)
10–19	20 (64.5)	11 (35.5)	0.6 (0.2–2.0)	0.6 (0.3–10.7)
20–24	4 (66.7)	2 (33.3)	0.7 (0.1–4.7)	1.0 (0.0–29.8)
25–34	16 (76.2)	5 (23.8)	1.1 (0.3–4.2)	0.7 (0.1–8.2)
≥ 35	25 (73.5)	9 (26.5)	1.0 (0.3–3.0)	0.7 (0.1–5.4)
**Area council**				
Abuja Municipal	65 (83.3)	13 (16.7)	ref	ref
Kuje	35 (85.4)	6 (14.6)	1.2 (0.4–3.3)	1.5 (0.4–5.6)
Kwali	7 (14.1)	22 (75.9)	0.1 (0.0–0.2.)*	0.1 (0.0–0.2)*
**Educational level**				
None	22 (81.5)	5 (18.5)	ref	ref
Primary	30 (71.4)	12 (28.6)	0.6 (0.2–1.2)	0.2 (0.0–1.5)
Secondary	29 (61.7)	18 (38.3)	0.4 (0.1–1.1)	0.1 (0.0–1.1)
Tertiary	26 (81.2)	6 (18.8)	1.0 (0.3–3.7)	0.6 (0.1–4.4)
**Slept in the same room with patient**				
Yes	59 (70.2)	25 (29.8)	0.8 (0.4–1.6)	1.6(0.6–4.3)
No	48 (75.0)	16 (25.0)	ref	ref
**Bushes around the house**				
Yes	34 (63.0)	20 (37.0)	0.5 (0.2–1.0)	0.4 (0.2–0.9)*
No	73 (77.7)	21 (22.3)	ref	ref
**Uncovered water receptacles around the house**				
Yes	22 (84.60)	4 (15.4)	2.4 (0.8–7.4)	1.9 (0.5–7.1)
No	85 (30.3)	37 (39.7)	ref	ref

*significant at 5% level; ref: reference category

The most common reason given (68.3%) for not using LLIN the night before the survey was “weather too hot”. Twenty-two (78.6%) of the 28 individuals who gave that response were from Kwali area council.

Of the 107 households, 102 (95.3%) had at least one household member with asymptomatic malaria parasitaemia. Of the 602 household members, 421 (69.9%, 95% CI: 66.2–73.5) had malaria parasitaemia and the median malaria parasite density for the positive malaria cases was 64 parasites/μl (IQR: 208 parasites/μL). The prevalence of malaria parasitaemia was 71.4% in Abuja Municipal, 63.4% in Kwali, and 68.2% in Kuje area councils. Malaria parasite infection cut across all age groups. However, it was highest among participants in the age group 5–9 years (80.4%) and lowest among participants who were 35 years and above (61.8%). Having bushes around the house (OR: 2.3, 95% CI: 1.6–3.5) was significantly associated with having asymptomatic malaria parasitaemia. Sleeping under LLIN the night before the survey was not significantly associated with having malaria parasitaemia in this study (OR: 0.9, 95% CI: 0.6–1.3). Having bushes around the house remained the only predictor of asymptomatic malaria parasitaemia after controlling for other variables (aOR: 2.7, 95% CI: 1.7–4.2) (Table 4).

**Table 4:** Predictors of asymptomatic malaria parasitaemia among household members of malaria patients, Abuja, Nigeria 2016 [N = 602].

Characteristics	Malaria parasite present N = 421	Malaria parasite absent N = 181	Crude OR (95% CI)	Adjusted OR (95% CI)
	n (%)	n (%)		
**Sex**				
Male	201 (74.2)	70 (25.8)	1.4 (1.0–2.1)	1.4 (1.0–2.0)
Female	220 (66.5)	111 (33.5)	ref	ref
**Age group (years)**				
<5	67 (73.6)	24 (26.4)	ref	ref
5–9	90 (80.4)	22 (19.6)	1.5 (0.8–2.8)	1.5 (0.8–2.9)
10–19	90 (66.2)	46 (33.8)	0.7 (0.4–1.3)	0.7 (0.4–1.3)
20–24	35 (70.0)	15 (30.0)	0.8 (0.4–1.8)	0.9 (0.4–2.0)
25–34	76 (68.5)	35 (31.5)	0.8 (0.4–1.4)	0.7 (0.4–1.4)
≥35	63 (61.8)	39 (38.2)	0.6 (0.3–1.1)	0.6 (0.3–1.0)
**Area council**				
Abuja municipal	110 (28.6)	275 (71.4)	ref	ref
Kuje	56 (31.8)	120 (68.2)	0.9 (0.6–1.3)	0.7 (0.4–1.2)
Kwali	15 (36.6)	26 (63.4)	0.7 (0.4–1.4)	0.7 (0.3–1.4)
**Slept in the same room with patient**				
Yes	190 (67.1)	93 (32.9)	0.8 (0.6–1.1)	0.8 (0.6–1.1)
No	231 (72.4)	88 (27.6)	ref	ref
**Uncovered water receptacles**				
Yes	52 (68.4)	24 (31.6)	0.9 (0.6–1.6)	0.7 (0.4–1.2)
No	369 (70.2)	157 (29.8)	ref	ref
**Bushes around house**				
Yes	169 (80.5)	41 (19.5)	2.3 (1.5–3.4)	2.7 (1.7–4.2) *
No	252 (64.3)	140 (35.7)	ref	ref
**LLIN use**				
Yes	72 (67.3)	35 (32.7)	0.9 (0.6–1.3)	1.0 (0.6–1.5)
No	349 (70.5)	146 (29.5)	ref	ref
**House walls**				
Mud	86 (68.3)	40 (31.7)	0.9 (0.6–1.4)	0.9 (0.5–1.5)
Cement plastered	335 (70.4)	141 (29.6)	ref	ref

*significant at 5% level;

## Discussion

This study investigates asymptomatic malaria parasitaemia prevalence and LLIN use among household members of malaria patients attending health facilities in FCT Abuja. The malaria parasitaemia prevalence of 69.9% found is higher than the prevalence of 36.6% found by Noland *et al*. (2014) in Plateau State, 63.7% found by Babamale and Ugbomoiko (2016) in Kwara State, and 60.7% found by Dawaki *et al*. (2016) in Kano State [10,15,16], all studies that were carried out among the general population in communities in Northern Nigeria. Studies targeting this population of households of index malaria patients were carried out only in areas of malaria elimination with low malaria prevalence [17,18].

The lack of association between LLIN use and malaria parasitaemia may be due to low ownership of LLIN in this study, thus insufficient coverage for community effect. Studies have found that community-level usage of bed nets is significantly associated with reduced risk of malaria [19,20] About 305,000 LLINs were distributed by mass campaign in the rural areas of FCT by NetMark, an international non-governmental organization (NGO) supported by USAID, in collaboration with the National Malaria Elimination Programme and three local NGOs from 2002 to 2009 [21]. Distribution channels included antenatal clinics, immunization and schools. Despite this, approximately three-quarters of our study participants did not own LLINs and most of the individuals who owned LLINs purchased them. This may be due to lack of awareness of where to get them free or nets given may have expired. This presents a need for increased awareness through the media or other channels on availability of LLINs and where to get them from. Despite having the highest proportion of LLIN owners, Kwali area council had the lowest proportion of LLIN use among those who owned LLINs. Use of LLIN among households that owned at least one LLIN was at least three times higher in AMAC/Kuje compared to Kwali. Hot temperature was given as a reason for not sleeping under the LLIN, which highlights a need for an additional intervention in that area during the hot weather. Low proportion of LLIN use in Kwali may have been responsible for higher malaria prevalence (4.8% and 8% more than Kuje and AMAC respectively) though this was not statistically significant. Long-lasting insecticidal nets have been found to be effective in preventing malaria [22–25] and cost-effective compared to other malaria prevention measures [26,27]. Those who had bushes around their homes were less likely to use LLINs and more likely to have malaria parasitaemia. Bushes serve as breeding places for mosquitoes and water droplets can be stored in big leaves. Studies in Ghana, Kenya, and Ethiopia have documented association between malaria and presence of vegetation [28–30].

The cross-sectional nature of this study may limit the causal and effect interpretation of the factors that are observed in the study. Information provided on LLIN use was self-reported and could not be verified. In interpreting the results, outdoor behavior of study participants was not considered. Prevalence of asymptomatic malaria recorded in this study using microscopy could have been higher if this was ascertained with polymerase chain reaction (PCR). However, PCR was not used in this study due to cost. Finally, few index cases were recruited in Kwali area council. However, the study was about household level transmission for which the index case provided a link and thus, the number of index cases recruited is unrelated at individual level.

## Conclusion

High prevalence of asymptomatic malaria parasitaemia and low use of LLIN among household members of malaria patients portend the risk of intra-household common source of malaria transmission. Predictors of LLIN use included having bushy surroundings and residing in Kwali area council. Having bushes around the house was an important predictor of malaria parasitaemia.

Environmental sanitation, clearing of bushes around the house, and scaling up of LLIN ownership and use by all age groups stand to reduce malaria transmission. Study to explore the role of preventive treatment of household members of confirmed malaria patient in curbing transmission is suggested. Malaria transmission may not be interrupted if these asymptomatic population are not identified and treated. Strategies promoting LLIN use need to be intensified in Kwali.
